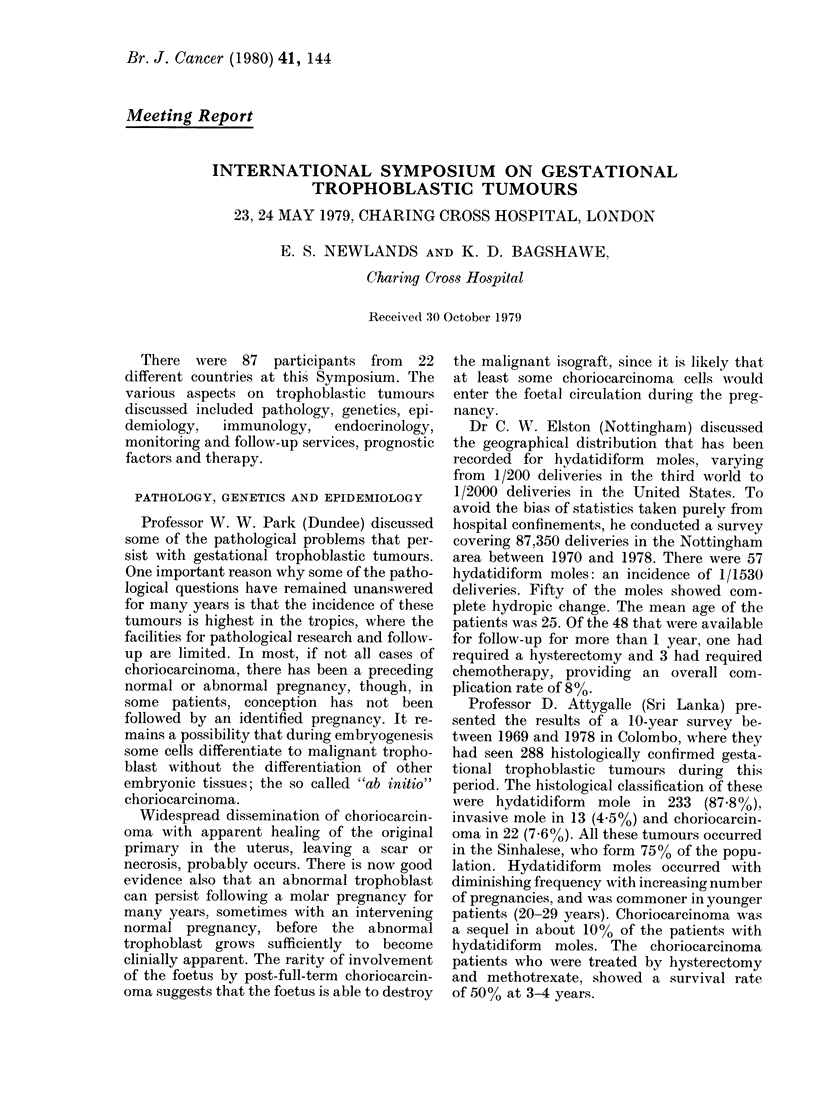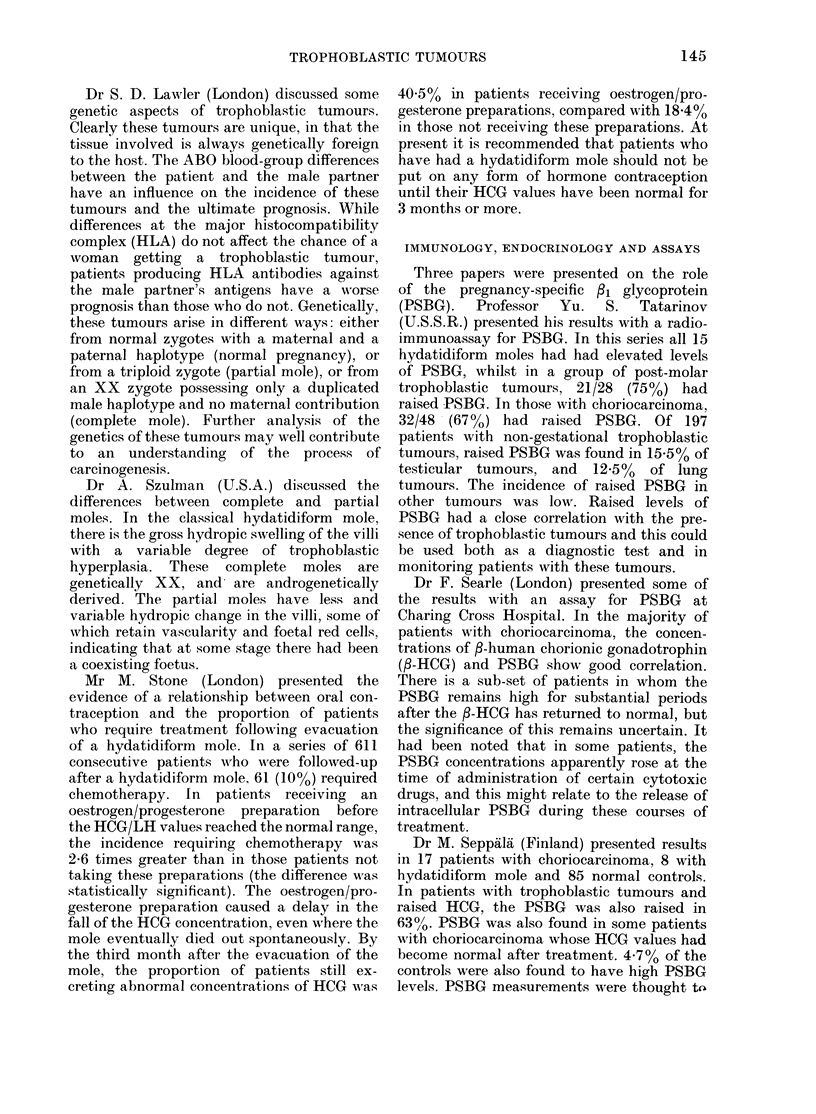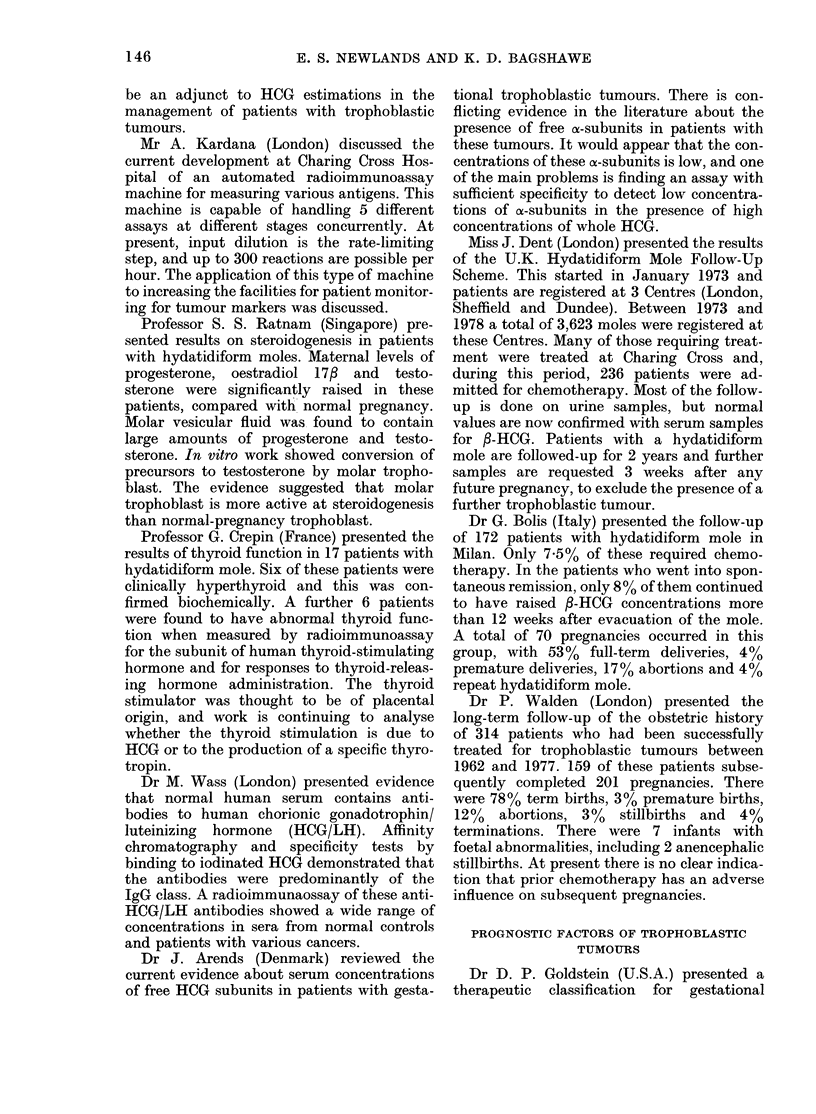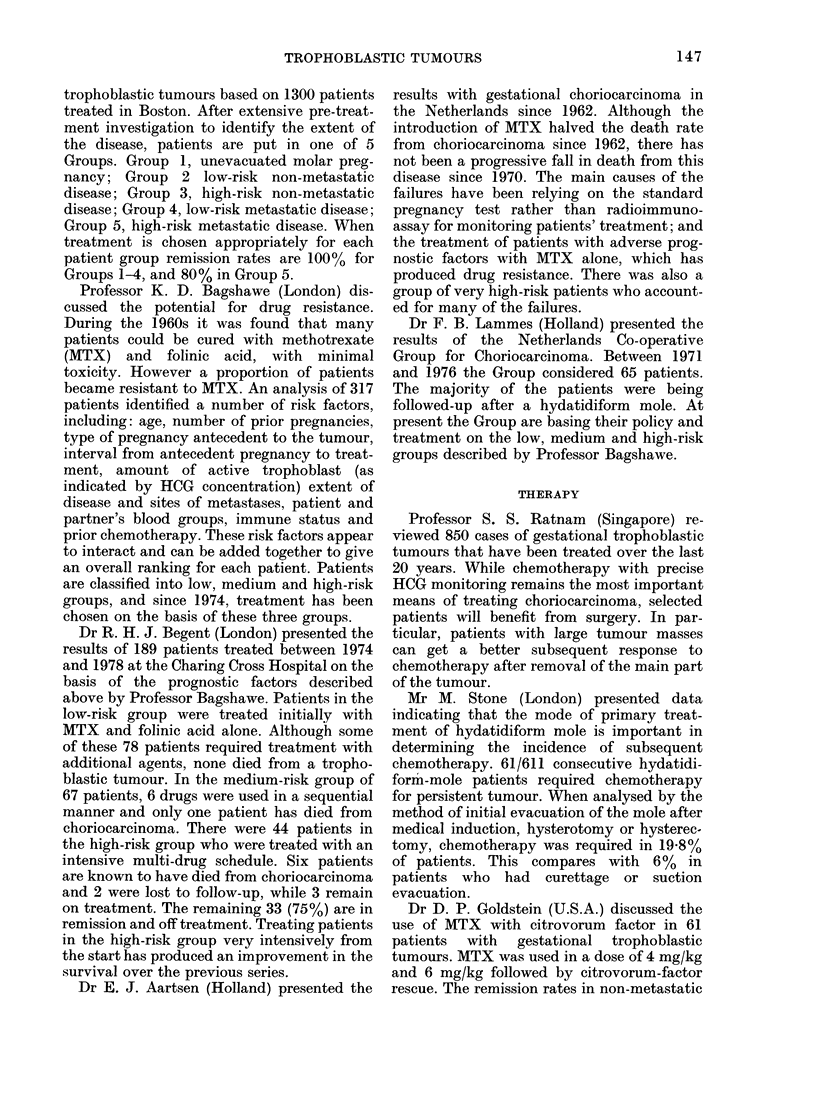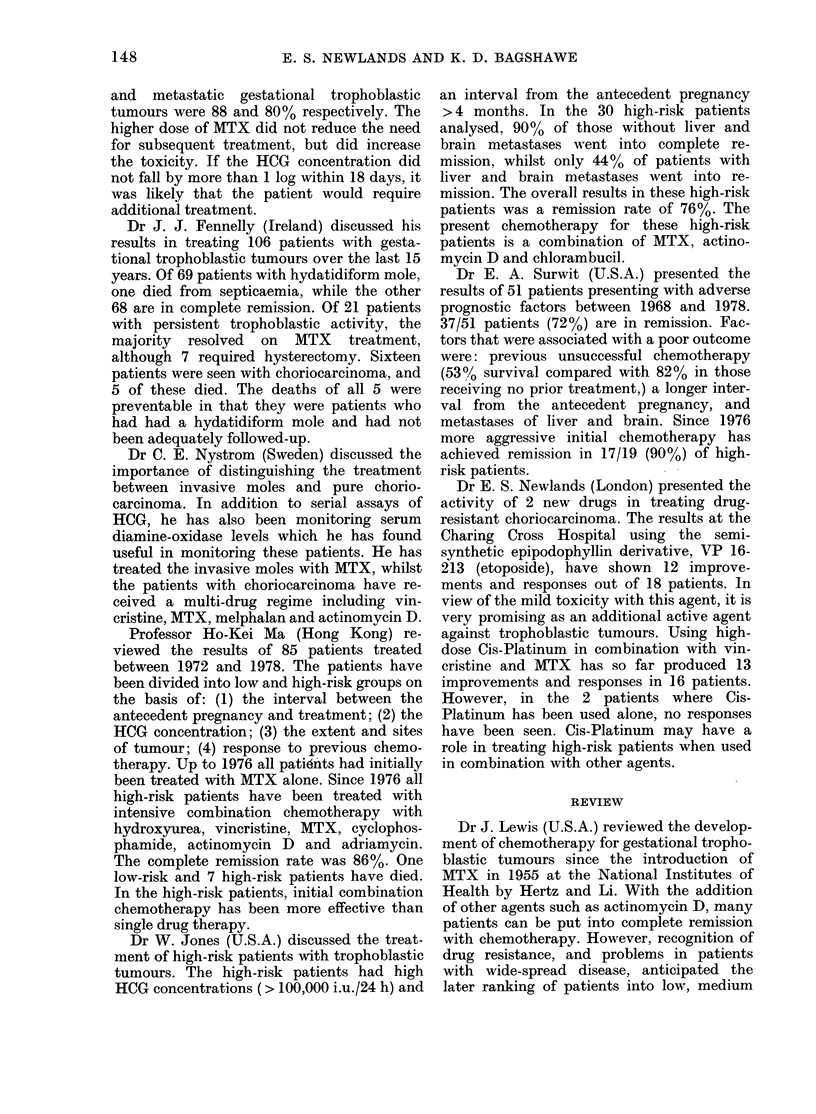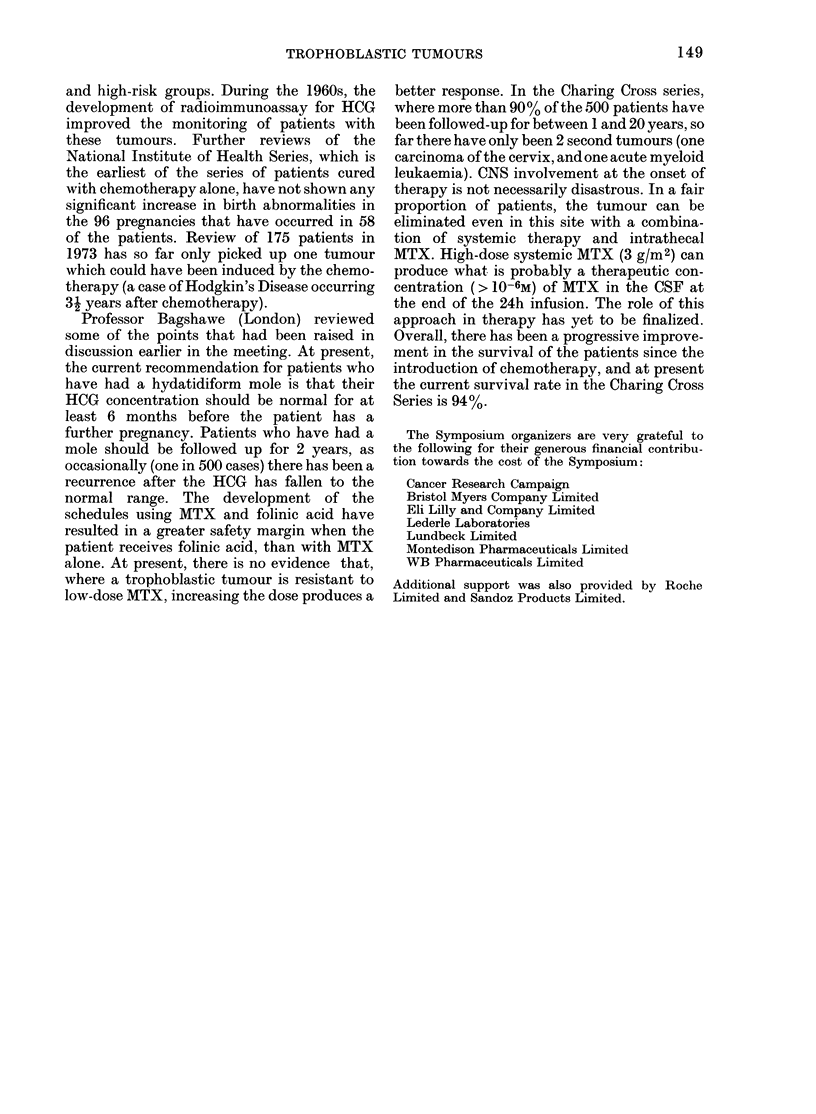# International symposium on gestational trophoblastic tumours

**Published:** 1980-01

**Authors:** E. S. Newlands, K. D. Bagshawe


*Br. J. Cancer* (1980) **41**, 144

### *Meeting Report*

### INTERNATIONAL SYMPOSIUM ON GESTATIONAL TROPHOBLASTIC TUMOURS

23, 24 MAY 1979, CHARING CROSS HOSPITAL, LONDON

E. S. NEWLANDS AND K. D. BAGSHAWE,

*Charing Cross Hospital*

Received 30 October 1979

There were 87 participants from 22 different countries at this Symposium. The various aspects on trophoblastic tumours discussed included pathology, genetics, epidemiology, immunology, endocrinology, monitoring and follow-up services, prognostic factors and therapy.

### PATHOLOGY, GENETICS AND EPIDEMIOLOGY

Professor W. W. Park (Dundee) discussed some of the pathological problems that persist with gestational trophoblastic tumours. One important reason why some of the pathological questions have remained unanswered for many years is that the incidence of these tumours is highest in the tropics, where the facilities for pathological research and follow-up are limited. In most, if not all cases of choriocarcinoma, there has been a preceding normal or abnormal pregnancy, though, in some patients, conception has not been followed by an identified pregnancy. It remains a possibility that during embryogenesis some cells differentiate to malignant trophoblast without the differentiation of other embryonic tissues; the so called "*ab initio*" choriocarcinoma.

Widespread dissemination of choriocarcinoma with apparent healing of the original primary in the uterus, leaving a scar or necrosis, probably occurs. There is now good evidence also that an abnormal trophoblast can persist following a molar pregnancy for many years, sometimes with an intervening normal pregnancy, before the abnormal trophoblast grows sufficiently to become clinially apparent. The rarity of involvement of the foetus by post-full-term choriocarcinoma suggests that the foetus is able to destroy the malignant isograft, since it is likely that at least some choriocarcinoma cells would enter the foetal circulation during the pregnancy.

Dr C. W. Elston (Nottingham) discussed the geographical distribution that has been recorded for hydatidiform moles, varying from 1/200 deliveries in the third world to 1/2000 deliveries in the United States. To avoid the bias of statistics taken purely from hospital confinements, he conducted a survey covering 87,350 deliveries in the Nottingham area between 1970 and 1978. There were 57 hydatidiform moles: an incidence of 1/1530 deliveries. Fifty of the moles showed complete hydropic change. The mean age of the patients was 25. Of the 48 that were available for follow-up for more than 1 year, one had required a hysterectomy and 3 had required chemotherapy, providing an overall complication rate of 8%.

Professor D. Attygalle (Sri Lanka) presented the results of a 10-year survey between 1969 and 1978 in Colombo, where they had seen 288 histologically confirmed gestational trophoblastic tumours during this period. The histological classification of these were hydatidiform mole in 233 (87·8%), invasive mole in 13 (4·5%) and choriocarcinoma in 22 (7·6%). All these tumours occurred in the Sinhalese, who form 75% of the population. Hydatidiform moles occurred with diminishing frequency with increasing number of pregnancies, and was commoner in younger patients (20–29 years). Choriocarcinoma was a sequel in about 10% of the patients with hydatidiform moles. The choriocarcinoma patients who were treated by hysterectomy and methotrexate, showed a survival rate of 50% at 3–4 years.

Dr S. D. Lawler (London) discussed some genetic aspects of trophoblastic tumours. Clearly these tumours are unique, in that the tissue involved is always genetically foreign to the host. The ABO blood-group differences between the patient and the male partner have an influence on the incidence of these tumours and the ultimate prognosis. While differences at the major histocompatibility complex (HLA) do not affect the chance of a woman getting a trophoblastic tumour, patients producing HLA antibodies against the male partner's antigens have a worse prognosis than those who do not. Genetically, these tumours arise in different ways: either from normal zygotes with a maternal and a paternal haplotype (normal pregnancy), or from a triploid zygote (partial mole), or from an XX zygote possessing only a duplicated male haplotype and no maternal contribution (complete mole). Further analysis of the genetics of these tumours may well contribute to an understanding of the process of carcinogenesis.

Dr A. Szulman (U.S.A.) discussed the differences between complete and partial moles. In the classical hydatidiform mole, there is the gross hydropic swelling of the villi with a variable degree of trophoblastic hyperplasia. These complete moles are genetically XX, and are androgenetically derived. The partial moles have less and variable hydropic change in the villi, some of which retain vascularity and foetal red cells, indicating that at some stage there had been a coexisting foetus.

Mr M. Stone (London) presented the evidence of a relationship between oral contraception and the proportion of patients who require treatment following evacuation of a hydatidiform mole. In a series of 611 consecutive patients who were followed-up after a hydatidiform mole, 61 (10%) required chemotherapy. In patients receiving an oestrogen/progesterone preparation before the HCG/LH values reached the normal range, the incidence requiring chemotherapy was 2·6 times greater than in those patients not taking these preparations (the difference was statistically significant). The oestrogen/progesterone preparation caused a delay in the fall of the HCG concentration, even where the mole eventually died out spontaneously. By the third month after the evacuation of the mole, the proportion of patients still excreting abnormal concentrations of HCG was 40·5% in patients receiving oestrogen/progesterone preparations, compared with 18·4% in those not receiving these preparations. At present it is recommended that patients who have had a hydatidiform mole should not be put on any form of hormone contraception until their HCG values have been normal for 3 months or more.

### IMMUNOLOGY, ENDOCRINOLOGY AND ASSAYS

Three papers were presented on the role of the pregnancy-specific $\beta_1$ glycoprotein (PSBG). Professor Yu. S. Tatarinov (U.S.S.R.) presented his results with a radioimmunoassay for PSBG. In this series all 15 hydatidiform moles had had elevated levels of PSBG, whilst in a group of post-molar trophoblastic tumours, 21/28 (75%) had raised PSBG. In those with choriocarcinoma, 32/48 (67%) had raised PSBG. Of 197 patients with non-gestational trophoblastic tumours, raised PSBG was found in 15·5% of testicular tumours, and 12·5% of lung tumours. The incidence of raised PSBG in other tumours was low. Raised levels of PSBG had a close correlation with the presence of trophoblastic tumours and this could be used both as a diagnostic test and in monitoring patients with these tumours.

Dr F. Searle (London) presented some of the results with an assay for PSBG at Charing Cross Hospital. In the majority of patients with choriocarcinoma, the concentrations of $\beta$-human chorionic gonadotrophin ($\beta$-HCG) and PSBG show good correlation. There is a sub-set of patients in whom the PSBG remains high for substantial periods after the $\beta$-HCG has returned to normal, but the significance of this remains uncertain. It had been noted that in some patients, the PSBG concentrations apparently rose at the time of administration of certain cytotoxic drugs, and this might relate to the release of intracellular PSBG during these courses of treatment.

Dr M. Seppälä (Finland) presented results in 17 patients with choriocarcinoma, 8 with hydatidiform mole and 85 normal controls. In patients with trophoblastic tumours and raised HCG, the PSBG was also raised in 63%. PSBG was also found in some patients with choriocarcinoma whose HCG values had become normal after treatment. 4·7% of the controls were also found to have high PSBG levels. PSBG measurements were thought to be an adjunct to HCG estimations in the management of patients with trophoblastic tumours.

Mr A. Kardana (London) discussed the current development at Charing Cross Hospital of an automated radioimmunoassay machine for measuring various antigens. This machine is capable of handling 5 different assays at different stages concurrently. At present, input dilution is the rate-limiting step, and up to 300 reactions are possible per hour. The application of this type of machine to increasing the facilities for patient monitoring for tumour markers was discussed.

Professor S. S. Ratnam (Singapore) presented results on steroidogenesis in patients with hydatidiform moles. Maternal levels of progesterone, oestradiol 17$\beta$ and testosterone were significantly raised in these patients, compared with normal pregnancy. Molar vesicular fluid was found to contain large amounts of progesterone and testosterone. *In vitro* work showed conversion of precursors to testosterone by molar trophoblast. The evidence suggested that molar trophoblast is more active at steroidogenesis than normal-pregnancy trophoblast.

Professor G. Crepin (France) presented the results of thyroid function in 17 patients with hydatidiform mole. Six of these patients were clinically hyperthyroid and this was confirmed biochemically. A further 6 patients were found to have abnormal thyroid function when measured by radioimmunoassay for the subunit of human thyroid-stimulating hormone and for responses to thyroid-releasing hormone administration. The thyroid stimulator was thought to be of placental origin, and work is continuing to analyse whether the thyroid stimulation is due to HCG or to the production of a specific thyrotropin.

Dr M. Wass (London) presented evidence that normal human serum contains antibodies to human chorionic gonadotrophin/luteinizing hormone (HCG/LH). Affinity chromatography and specificity tests by binding to iodinated HCG demonstrated that the antibodies were predominantly of the IgG class. A radioimmunaoassay of these anti-HCG/LH antibodies showed a wide range of concentrations in sera from normal controls and patients with various cancers.

Dr J. Arends (Denmark) reviewed the current evidence about serum concentrations of free HCG subunits in patients with gestational trophoblastic tumours. There is conflicting evidence in the literature about the presence of free $\alpha$-subunits in patients with these tumours. It would appear that the concentrations of these $\alpha$-subunits is low, and one of the main problems is finding an assay with sufficient specificity to detect low concentrations of $\alpha$-subunits in the presence of high concentrations of whole HCG.

Miss J. Dent (London) presented the results of the U.K. Hydatidiform Mole Follow-Up Scheme. This started in January 1973 and patients are registered at 3 Centres (London, Sheffield and Dundee). Between 1973 and 1978 a total of 3,623 moles were registered at these Centres. Many of those requiring treatment were treated at Charing Cross and, during this period, 236 patients were admitted for chemotherapy. Most of the follow-up is done on urine samples, but normal values are now confirmed with serum samples for $\beta$-HCG. Patients with a hydatidiform mole are followed-up for 2 years and further samples are requested 3 weeks after any future pregnancy, to exclude the presence of a further trophoblastic tumour.

Dr G. Bolis (Italy) presented the follow-up of 172 patients with hydatidiform mole in Milan. Only 7·5% of these required chemotherapy. In the patients who went into spontaneous remission, only 8% of them continued to have raised $\beta$-HCG concentrations more than 12 weeks after evacuation of the mole. A total of 70 pregnancies occurred in this group, with 53% full-term deliveries, 4% premature deliveries, 17% abortions and 4% repeat hydatidiform mole.

Dr P. Walden (London) presented the long-term follow-up of the obstetric history of 314 patients who had been successfully treated for trophoblastic tumours between 1962 and 1977. 159 of these patients subsequently completed 201 pregnancies. There were 78% term births, 3% premature births, 12% abortions, 3% stillbirths and 4% terminations. There were 7 infants with foetal abnormalities, including 2 anencephalic stillbirths. At present there is no clear indication that prior chemotherapy has an adverse influence on subsequent pregnancies.

### PROGNOSTIC FACTORS OF TROPHOBLASTIC TUMOURS

Dr D. P. Goldstein (U.S.A.) presented a therapeutic classification for gestational trophoblastic tumours based on 1300 patients treated in Boston. After extensive pre-treatment investigation to identify the extent of the disease, patients are put in one of 5 Groups. Group 1, unevacuated molar pregnancy; Group 2 low-risk non-metastatic disease; Group 3, high-risk non-metastatic disease; Group 4, low-risk metastatic disease; Group 5, high-risk metastatic disease. When treatment is chosen appropriately for each patient group remission rates are 100% for Groups 1–4, and 80% in Group 5.

Professor K. D. Bagshawe (London) discussed the potential for drug resistance. During the 1960s it was found that many patients could be cured with methotrexate (MTX) and folinic acid, with minimal toxicity. However a proportion of patients became resistant to MTX. An analysis of 317 patients identified a number of risk factors, including: age, number of prior pregnancies, type of pregnancy antecedent to the tumour, interval from antecedent pregnancy to treatment, amount of active trophoblast (as indicated by HCG concentration) extent of disease and sites of metastases, patient and partner's blood groups, immune status and prior chemotherapy. These risk factors appear to interact and can be added together to give an overall ranking for each patient. Patients are classified into low, medium and high-risk groups, and since 1974, treatment has been chosen on the basis of these three groups.

Dr R. H. J. Begent (London) presented the results of 189 patients treated between 1974 and 1978 at the Charing Cross Hospital on the basis of the prognostic factors described above by Professor Bagshawe. Patients in the low-risk group were treated initially with MTX and folinic acid alone. Although some of these 78 patients required treatment with additional agents, none died from a trophoblastic tumour. In the medium-risk group of 67 patients, 6 drugs were used in a sequential manner and only one patient has died from choriocarcinoma. There were 44 patients in the high-risk group who were treated with an intensive multi-drug schedule. Six patients are known to have died from choriocarcinoma and 2 were lost to follow-up, while 3 remain on treatment. The remaining 33 (75%) are in remission and off treatment. Treating patients in the high-risk group very intensively from the start has produced an improvement in the survival over the previous series.

Dr E. J. Aartsen (Holland) presented the results with gestational choriocarcinoma in the Netherlands since 1962. Although the introduction of MTX halved the death rate from choriocarcinoma since 1962, there has not been a progressive fall in death from this disease since 1970. The main causes of the failures have been relying on the standard pregnancy test rather than radioimmunoassay for monitoring patients' treatment; and the treatment of patients with adverse prognostic factors with MTX alone, which has produced drug resistance. There was also a group of very high-risk patients who accounted for many of the failures.

Dr F. B. Lammes (Holland) presented the results of the Netherlands Co-operative Group for Choriocarcinoma. Between 1971 and 1976 the Group considered 65 patients. The majority of the patients were being followed-up after a hydatidiform mole. At present the Group are basing their policy and treatment on the low, medium and high-risk groups described by Professor Bagshawe.

### THERAPY

Professor S. S. Ratnam (Singapore) reviewed 850 cases of gestational trophoblastic tumours that have been treated over the last 20 years. While chemotherapy with precise HCG monitoring remains the most important means of treating choriocarcinoma, selected patients will benefit from surgery. In particular, patients with large tumour masses can get a better subsequent response to chemotherapy after removal of the main part of the tumour.

Mr M. Stone (London) presented data indicating that the mode of primary treatment of hydatidiform mole is important in determining the incidence of subsequent chemotherapy. 61/611 consecutive hydatidiform-mole patients required chemotherapy for persistent tumour. When analysed by the method of initial evacuation of the mole after medical induction, hysterotomy or hysterectomy, chemotherapy was required in 19·8% of patients. This compares with 6% in patients who had curettage or suction evacuation.

Dr D. P. Goldstein (U.S.A.) discussed the use of MTX with citrovorum factor in 61 patients with gestational trophoblastic tumours. MTX was used in a dose of 4 mg/kg and 6 mg/kg followed by citrovorum-factor rescue. The remission rates in non-metastatic and metastatic gestational trophoblastic tumours were 88 and 80% respectively. The higher dose of MTX did not reduce the need for subsequent treatment, but did increase the toxicity. If the HCG concentration did not fall by more than 1 log within 18 days, it was likely that the patient would require additional treatment.

Dr J. J. Fennelly (Ireland) discussed his results in treating 106 patients with gestational trophoblastic tumours over the last 15 years. Of 69 patients with hydatidiform mole, one died from septicaemia, while the other 68 are in complete remission. Of 21 patients with persistent trophoblastic activity, the majority resolved on MTX treatment, although 7 required hysterectomy. Sixteen patients were seen with choriocarcinoma, and 5 of these died. The deaths of all 5 were preventable in that they were patients who had had a hydatidiform mole and had not been adequately followed-up.

Dr C. E. Nystrom (Sweden) discussed the importance of distinguishing the treatment between invasive moles and pure choriocarcinoma. In addition to serial assays of HCG, he has also been monitoring serum diamine-oxidase levels which he has found useful in monitoring these patients. He has treated the invasive moles with MTX, whilst the patients with choriocarcinoma have received a multi-drug regime including vincristine, MTX, melphalan and actinomycin D.

Professor Ho-Kei Ma (Hong Kong) reviewed the results of 85 patients treated between 1972 and 1978. The patients have been divided into low and high-risk groups on the basis of: (1) the interval between the antecedent pregnancy and treatment; (2) the HCG concentration; (3) the extent and sites of tumour; (4) response to previous chemotherapy. Up to 1976 all patients had initially been treated with MTX alone. Since 1976 all high-risk patients have been treated with intensive combination chemotherapy with hydroxyurea, vincristine, MTX, cyclophosphamide, actinomycin D and adriamycin. The complete remission rate was 86%. One low-risk and 7 high-risk patients have died. In the high-risk patients, initial combination chemotherapy has been more effective than single drug therapy.

Dr W. Jones (U.S.A.) discussed the treatment of high-risk patients with trophoblastic tumours. The high-risk patients had high HCG concentrations ( > 100,000 i.u./24 h) and an interval from the antecedent pregnancy >4 months. In the 30 high-risk patients analysed, 90% of those without liver and brain metastases went into complete remission, whilst only 44% of patients with liver and brain metastases went into remission. The overall results in these high-risk patients was a remission rate of 76%. The present chemotherapy for these high-risk patients is a combination of MTX, actinomycin D and chlorambucil.

Dr E. A. Surwit (U.S.A.) presented the results of 51 patients presenting with adverse prognostic factors between 1968 and 1978. 37/51 patients (72%) are in remission. Factors that were associated with a poor outcome were: previous unsuccessful chemotherapy (53% survival compared with 82% in those receiving no prior treatment,) a longer interval from the antecedent pregnancy, and metastases of liver and brain. Since 1976 more aggressive initial chemotherapy has achieved remission in 17/19 (90%) of high-risk patients.

Dr E. S. Newlands (London) presented the activity of 2 new drugs in treating drug-resistant choriocarcinoma. The results at the Charing Cross Hospital using the semi-synthetic epipodophyllin derivative, VP 16-213 (etoposide), have shown 12 improvements and responses out of 18 patients. In view of the mild toxicity with this agent, it is very promising as an additional active agent against trophoblastic tumours. Using high-dose Cis-Platinum in combination with vincristine and MTX has so far produced 13 improvements and responses in 16 patients. However, in the 2 patients where Cis-Platinum has been used alone, no responses have been seen. Cis-Platinum may have a role in treating high-risk patients when used in combination with other agents.

### REVIEW

Dr J. Lewis (U.S.A.) reviewed the development of chemotherapy for gestational trophoblastic tumours since the introduction of MTX in 1955 at the National Institutes of Health by Hertz and Li. With the addition of other agents such as actinomycin D, many patients can be put into complete remission with chemotherapy. However, recognition of drug resistance, and problems in patients with wide-spread disease, anticipated the later ranking of patients into low, medium and high-risk groups. During the 1960s, the development of radioimmunoassay for HCG improved the monitoring of patients with these tumours. Further reviews of the National Institute of Health Series, which is the earliest of the series of patients cured with chemotherapy alone, have not shown any significant increase in birth abnormalities in the 96 pregnancies that have occurred in 58 of the patients. Review of 175 patients in 1973 has so far only picked up one tumour which could have been induced by the chemotherapy (a case of Hodgkin's Disease occurring 3½ years after chemotherapy).

Professor Bagshawe (London) reviewed some of the points that had been raised in discussion earlier in the meeting. At present, the current recommendation for patients who have had a hydatidiform mole is that their HCG concentration should be normal for at least 6 months before the patient has a further pregnancy. Patients who have had a mole should be followed up for 2 years, as occasionally (one in 500 cases) there has been a recurrence after the HCG has fallen to the normal range. The development of the schedules using MTX and folinic acid have resulted in a greater safety margin when the patient receives folinic acid, than with MTX alone. At present, there is no evidence that, where a trophoblastic tumour is resistant to low-dose MTX, increasing the dose produces a better response. In the Charing Cross series, where more than 90% of the 500 patients have been followed-up for between 1 and 20 years, so far there have only been 2 second tumours (one carcinoma of the cervix, and one acute myeloid leukaemia). CNS involvement at the onset of therapy is not necessarily disastrous. In a fair proportion of patients, the tumour can be eliminated even in this site with a combination of systemic therapy and intrathecal MTX. High-dose systemic MTX (3 g/m$^2$) can produce what is probably a therapeutic concentration ($>10^{-6}$M) of MTX in the CSF at the end of the 24h infusion. The role of this approach in therapy has yet to be finalized. Overall, there has been a progressive improvement in the survival of the patients since the introduction of chemotherapy, and at present the current survival rate in the Charing Cross Series is 94%.

The Symposium organizers are very grateful to the following for their generous financial contribution towards the cost of the Symposium:

Cancer Research Campaign
Bristol Myers Company Limited
Eli Lilly and Company Limited
Lederle Laboratories
Lundbeck Limited
Montedison Pharmaceuticals Limited
WB Pharmaceuticals Limited

Additional support was also provided by Roche Limited and Sandoz Products Limited.